# Being Called “Elderly” Impacts Adult Development: A Critical Analysis of Enduring Ageism During COVID in NZ Online News Media

**DOI:** 10.1007/s10804-022-09405-8

**Published:** 2022-05-25

**Authors:** Diana Amundsen

**Affiliations:** School of Education, University of Waikato, 101 Durham St, Tauranga, 3110 New Zealand

**Keywords:** Ageism, Online news media, Older adults, Elderly, Critical gerontological framing analysis, Adult development

## Abstract

This article examines how “the elderly” is constructed in New Zealand online news media. By employing a critical framing analysis to challenge ageist practices, conceptually, the study adds to our knowledge of research methodologies in the field of adult development. Online news media articles were collected and analyzed to understand constructions of older adults as “elderly” over an 18-month period before, during, and since the COVID pandemic. Results demonstrated that the term “elderly” was framed powerlessly, in predominantly negative (74%) stereotypical messages about older adults. Positive stereotypes (26% of data) used human impact framing. Associations of “elderly” with being vulnerable, declining, and an individual or societal burden have serious implications, notably for the media in their role of both constructing and reflecting societal attitudes and actions towards older adults. Suggestions are offered to encourage reframing societal attitudes and promoting healthy adult development through age-equality messages that do away with the term “elderly.”

## Introduction

Language depicting older adults can be powerful for promoting critical thinking and supporting agency for social change, or conversely, for connoting discrimination and negative stereotypes. Research has shown that the term “elderly” applied to older adults is linked to negative stereotyping, discrimination, and ageism towards people during their later life development (Avers et al., 2011; Mautner, 2007). Effects of ageism for corroding healthy development among older adults are also becoming more widely discussed (United Nations [UN], 2021). Being labeled “elderly” associates with meanings of vulnerability, disability, and disempowerment (Mautner, 2007), which in turn, may link to ineffective coping or social isolation and loneliness in older adulthood.

Less is known about how everyday usage of this term “elderly,” in social or public spaces, influences older adult development. Scholars have identified news media articles as appropriate sources for exploring how language meanings and assumptions are societally constructed (Morgan et al., 2021; Vassil & Wass, 2006). News articles are widely consumed by diverse groups of adults (Wiles et al., 2012) and continue to represent a substantial element among New Zealand’s social environment. From the outset of the COVID pandemic beginning in 2020, media coverage of “elderly” was heightened in New Zealand (and likely elsewhere too).

In New Zealand, Prime Minister Jacinda Ardern made a “go hard and early” COVID response and quickly shut national borders. Creating a culture of compliance, stringent national lockdown guidelines began in March 2020 which recommended citizens stay home other than for essential personal movement. For people over 70 years, lockdown began earlier, and finished later. Subsequent research (Morgan et al., 2021) is emerging about the serious impacts of prolonged social isolation and harmful effects for older people who were most impacted by these policies. Morgan et al.’s (2021) qualitative document analysis study of 91 articles found that coverage stigmatized “elderly” as passive, vulnerable, unable to navigate threats to their health, or respond effectively to the move to digitalization (e.g., ordering groceries online, undertaking online banking during strict lockdowns).

The present research was conducted before and during the early onset of COVID at a time which affected older adults perhaps more than any other age groups. Given that the term “elderly” is evidently in circulation within everyday New Zealand society, the purpose of the study was to understand how language meanings and assumptions (Gendron et al., 2016) of “elderly” were characterized in online news media in New Zealand and determine impacts for older adult development.

### “Elderly”

Debate over whether “elderly” is polite or offensive has previously attracted research interest. Mautner (2007) proposed that the term “elderly” is a key expression for communication about older adults and aging, but its associative meaning is contested. Her etymological research established that “elderly” traces back to an ageist collocational profile with negative connotations including disability, illness, care, and vulnerability to crime. When looking for associations of “independence, initiative, and empowerment” Mautner (2007, p.63) found they were non-existent. Further, negativity compounds when “the elderly” is used as a collective expression, portraying a passive group, dependent on care, and victims of crime. In short, “elderly” emerges less as a marker of chronological age, than of perceived social consequences, negatively framed.

Lacking specificity, “elderly” connotes generalizations and assumptions of older adults as a homogenous group. In 1991, use of the term “older persons” was recommended in the *United Nations Principles for Older Persons* (UN, 1991). Twenty years on, researchers (Avers et al., 2011) revealed continuing usage of the term “elderly,” yet still concluded that the term is ageist. Despite outright rejection and protests by older adults themselves of this disparaging term (Avers et al., 2011; Mather, 2021; UN, 1991), use of “elderly” persists widely.

New Zealand is a predominantly western country located in the Pacific Ocean with a population of approximately 5.1 million. Around 819,000 (17%) are aged over 65 years (StatsNZ, 2021). Recent research in New Zealand underscores a growing opposition among older adults to the term “elderly” (Amundsen, 2021a, 2021b, 2021c, 2021d, 2022a, 2022b; Clement, 2021). Mather (2021, p. 1) reports leading New Zealand gerontologist Dame Peggy Koopman-Boyden calling for change: “Don’t call us elderly!” In response to an increase in national policies aiming to address aging, health, and wellbeing inequities (Parr-Brownlie et al., 2020), a deconstruction of this persistent form of ageism in public media is long overdue to learn how aging is constructed during adulthood within national identities.

### Reframing Aging

New Zealand, like other countries, is guided by the United Nations (UN) in promoting and encouraging respect for human rights, and fundamental freedoms without distinction as to race, sex, language, religion, or age. The *UN Decade of Healthy Aging 2021–2030s* (UN, 2021) four action areas are as directly relevant to New Zealanders as they are globally, notably the goal of combatting ageism through aiming to change narratives around age and aging. For decades, scholars of ageist discourses have been guided by Butler’s (1969, 1980) work explaining ageism as systemic attitudes and practices of stereotyping and discrimination towards people because they are old.

Taking up this enduring ageism issue, the *Global Report on Ageism* (World Health Organization [WHO], 2021) reflects contemporary understandings of how ageism influences our thoughts, feelings, and actions towards others and ourselves based on age. The report identifies how widespread ageism is globally, (across institutions, laws, policies) but worse, how damaging ageism is to individual health and dignity through its denial of human rights that individuals may reach their full potential. “Among older people, ageism is associated with poorer physical and mental health, increased social isolation and loneliness, greater financial insecurity, decreased quality of life and premature death” (WHO, 2021, p. ix). Over the lifespan, a compounding disadvantage can be seen when ageism intersects with ableism, sexism, and racism.

Beyond New Zealand, encouraging initiatives are emerging to reframe aging. The US *Reframing Aging* project, established in 2012, is a long-term social change initiative aiming to improve public knowledge of what aging means, and how older citizens contribute to society. Their useful style guide has been taken up by, “thousands of scholars, researchers, communications professionals, journalists, students, and others to adopt bias-free language and incorporate the principles of reframing aging” (Reframing Aging Initiative, 2022, p. 1). Similarly, the UK’s *Centre for Ageing Better* (Centre for Aging Better.org, 2022) aims to use research-informed evidence to change public perceptions and narratives of old age and aging. They have also produced guidance for language and writing about aging for the Independent Press Standards Organisation (IPSO)’s resources for journalists. Such initiatives lag in New Zealand; this study also intended to contribute to wider global efforts of reframing aging.

### Critical Gerontology and Framing Methodology

Critical gerontological theories underpinned the research process. In alignment with critical gerontological studies (Katz, 2019; Stephens, 2017; Wellin, 2018; Wiles et al., 2012), this study’s conceptual framework aimed to challenge the status quo and critique assumptions underpinning the biomedical model of aging while advocating for social understandings of how older adults can purposefully and resiliently navigate their lives.

Critical framing blends critical gerontological approaches with traditional media framing analysis. Concepts of framing began with Goffman (1974) to label schemata of interpretation. Entman (1993) later advanced framing analysis into a substantial methodological approach which researchers have continued applying to understand how print and other media present information.

Media framing typically reveals itself through the choice of key words or phrases to reinforce certain representations of reality (and emotions towards it). Media scholars (Altheide & Schneider, 2013; Stone, 2012) compare frames to boundaries which obscure our view or block off one thing while drawing our attention to something else. Linström and Marais (2012) similarly apply the idea of framing to “explain how the media structure their delivery of news, promoting certain interpretations of events by selecting certain facts” (p. 21).

Entman’s (1993) vision of traditional media frame analysis serves four main purposes: (a) define problems; (b) diagnose a course; (c) make value judgments; (d) suggest remedies. Importantly for this research, a *critical* framing analysis serves these four purposes *with an intention of* deconstructing assumptions underpinning the issue being framed and advocating for social change to occur through media influence. The real value of a critical framing analysis was not just as a reliable research method, but also as a provocative examination to contest the current situation and stir resolve for social change. The present study was therefore designed as a critical gerontological framing analysis of “elderly” to understand how old age is framed in online media text in New Zealand.

## Media Depictions of Aging

Media depictions of aging and older adulthood are attracting interest across diverse fields, for example, cultural gerontology (Wellin, 2018; Ylänne, 2021), media and communications (Ayalon et al., 2020; Sedick & Roost, 2011; Xi et al., 2020), political science (Zhuravskaya et al., 2020), and health and wellbeing (Avers et al., 2011; Jiao & Chang, 2020; Robinson & Callister, 2008). This body of research documents how media influences cultural attitudes, norms, and values; plays a key role in discourses and stereotypes of old age and promotes a cultural or social imaginary of later life (Ayalon et al., 2020; Dahmen & Cozma, 2009; Sedick & Roos, 2011; Ylänne, 2021). Media portrayal of aging affects development for adults of all ages, not just older adults themselves.

Research about media depictions of aging clearly demonstrates that older adults are under-represented and frequently absent in typical media forms like advertising (Ylänne, 2021), television, film, and print (Edström, 2018; Hurd et al., 2020). Studies purely focused on news articles, although rare, are more emerging (Hurd et al., 2020; Koskinen et al., 2014; Kovács et al., 2021). In both non-western and western countries, representation proportions of older adults in news media and commercials are dramatically less than actual population numbers (Edström, 2018; Prieler et al., 2016).

Sedick and Roos (2011) found that negative stereotyping of aging not only affects older citizens, but it also drives a wedge between intergenerational relations. Zeng and Abidin (2021) discuss how the “intergenerational politics” (p. 2459) between “boomers” and “zoomers” creates a powerful framing of public debates concerning inequality and economic polarization between “wealth-hoarding boomers” and “wage-frittering millennials.”

Individually, people are likely to reinforce, confirm, and develop prevalent attitudes, beliefs, and actions to match those presented in the media. Gerbner (1998) explained this phenomenon as *cultivation theory* when examining influences of television on viewers. Cultivation theory posits that over time, media exposure shapes how consumers perceive the world and conduct themselves—they gradually cultivate a view of reality closer to media’s depiction of reality. In this regard, media portrayals of aging can have powerful psychological impacts on individuals and generational cohorts of adults who gradually internalize and individually believe the stereotypes (Robinson & Callister, 2008; Vasil & Wass, 2006).

All these literature amount to a consensus that media depictions influence cultural attitudes and reflect societal norms of acceptability about aging. Continued ageism within news media occurs *because* it reflects cultural norms and beliefs, otherwise it would not be considered socially acceptable (as it is, largely). Assumptions that media alone drives and influences narratives and attitudes about “the elderly” would not be rational, but similarly, it would be a mistake to overlook the media’s role in constructing age-related discourses.

Similarities are likely between traditional print media and internet-based online media of older adult discourses. Makita et al. (2021) contend that although studies are emerging about online—including social media—representations of aging and older adults, there are still few. Given the enormity of online news media communication in current society, this is a significant omission which this research intends to address.

### Contradictory Stereotypes of Aging

When it comes to research on media stereotypes of older adults, literature is contradictory. Some studies have found that media depictions reinforce ageist stereotypes by characterizing the later life period as inevitable decline, frailty, poor health, senility, dependency, and obsolescence (see Hurd et al., 2020). Print news media studies also report rendering older people as a family burden, and societal drain on health care and social service organizations (Hurd et al., 2020; Rozanova et al., 2016).

However, in tension with these more negative stereotypical portrayals, other research suggests that, increasingly, media stereotypes construct later life as being possible (even ‘normal’) to have youthful agency, health, wealth, leisure, and be sexually, socially, and bodily able (Marshall & Rahman, 2015). Katz (2005) has suggested that media stereotypes depict the older adulthood life stage as “growing older without aging” (p. 188)—a kind of “agelessism” (Jiao & Chang, 2020, p. 571), while Sandberg and Marshall (2017) found prevalent stereotypes of aging as being “hetero-happy” (p. 3). Such messages reproduce a stereotype that aging is a personal choice; a product of lifestyle behaviors; and that a happy long life is positioned in relation to heterosexual coupledom, children, and grandchildren.

Apparent contradictions in research findings become less so if we interpret the representation to employ negative versus positive stereotypes, therefore constructing ageist portrayals overall (just of diverse types). In this sense, the term “elderly” might be studied in terms of its alignment with negative or positive stereotypes, and it would be reasonable to expect the term to have negative connotations if considering the etymological background for the term “elderly” established by Mautner (2007) discussed earlier. Given widespread use of the term “the elderly” in New Zealand, this research set out to understand how the “elderly” are characterized in online news media in New Zealand.

## Method

### Design

Ethical approval (Amundsen & Msoroka, 2019) was not required to conduct this study as news media is widely available publicly. Within critical framing analysis approaches, word choice is one device that helps to establish media frames (Linström & Marais, 2012). Search terms were “elderly” and “the elderly.” Articles obtained published between 01 January 2019 and 30 June 2020 were retrieved for analysis from the Newztext database. This period was significant, as the COVID pandemic first broke out during data collection. Linström and Marais (2012) articulate a method for identifying news frames in *print* media, which was adopted for this *online* media study to critically analyze stereotypes of “elderly” in a New Zealand context.

The search included articles published in English, where the search term could appear in either the headline or the article itself. Target sources included three major New Zealand media groups: Fairfax Metropolitan and Provincial Newspapers (10), Herald (10) and Other (7), including Daily Newswires, totaling 27 outlets. These media outlets were investigated because they comprise the main (though not quite all) online text-based news outlets in New Zealand, and represent a wide range of news publications nationally, regionally, across geographically diverse regions.

### Data

Significant amounts of data were retrieved from the Newztext database and systematically placed in the project MS Excel spreadsheet. All items were screened for relevance. Duplicates were removed; remaining data were transferred into the project MS Excel spreadsheet ready for framing analysis. A different worksheet was used for each month (*N* = *18* worksheets). Each worksheet was organized with columns containing data such as the article date, source, headline, phrase(s) containing the search term and any other relevant information like story topic, story type i.e., opinion piece, feature article, report, letter to the editor, regional or national news. In all, a total of 6690 phrases containing “the elderly” comprised the data set (Appendix 1 provides a data extract, accompanied by corresponding numbers to indicate the origins of quotes that will later feature in the findings section of this article).

### Procedure

First, phrases in worksheets 1–3 (January to March 2019) were read to establish provisional frames and core concepts using operational definitions (Linström & Marais, 2012). Then, three more worksheets (4–6; April to June 2019) were read to further refine frames. Starting from a hypothesis that some of the references to “the elderly” were more relevant to the core of the frames than others, certain phrases become marker phrases. Article headlines were another powerful indicator of the frame of the story or phrase. Following this, strongly relevant phrases among the six sheets of data were used to evaluate and consolidate these ideas in the form of initial codes. During this iterative process, note-taking around how ideas were generated maximized transparency and ensured that the frames and codes were closely derived from the data (Denzin & Lincoln, 2011). Once this groundwork was laid, the remainder of the worksheets (7–18) were read and coded.

Yet, a third in-depth interpretation of the phrases, the ‘what’ and ‘how’ of the frame, took place. During the third in-depth reading, phrases were categorized to describe aspects of the phrase, including the story topics, pairing with other keywords, values, and specific stereotypes. As part of this process, codes that were like each other were gradually organized into more general, higher-level codes (which became stereotype categories). Codes could also be attached to more than one stereotype category, making possible a multi-dimensional grouping of codes. For example, higher-level codes/stereotype categories of ‘demographic time-bomb,’ ‘societal drain,’ ‘non-contributor,’ ‘past-it,’ ‘obsolete,’ and ‘family problem’ were combined to form an overarching category of ‘burden.’ Each overarching category was cross-checked to the operational definitions of the dominant and secondary frames.

Ultimately, for each phrase key elements were identified: the main story topic; whether “the elderly” were framed using negative or positive stereotypes; whether the emphasis of the phrase (or article if it was necessary to understand the wider context in which the phrase was used) was on an individual or a societal/collective narrative; how “the elderly” was positioned in response to COVID; and what was the take-home message. In this sense, readers may see overlaps with a thematic analysis (Braun & Clarke, 2006). Here, it is important to reiterate that frames are the media’s perspectives, whereas themes are researchers’ interpretation of expressed products of those framed perspectives. As Goffman (1974) originally envisaged, frames are used to label schemata of interpretation and in this study, the framing analysis pinpointed stereotype categories as the schemata to interpret the media’s conceptual origins of inclusion or exclusion. Results are now presented with support from direct excerpts of phrases.

## Results

Overall, almost three-quarters of all data (74%) were framed using negative stereotypes and approximately one-quarter (26%) were framed with positive stereotypes of “the elderly.” Predominantly, the powerlessness frame was used for three negative categories of vulnerable (34%), declining (21%), and burden (19%), whereas the human impact frame was used for three positive categories of mentor or role model (17%), perfect grandparent (5%), and golden oldie (4%) as illustrated in Table 1.

**Table 1:** Frames, categories, and stereotypes percentages

Negative framing 74%		Positive framing 26%	
Frame and category	Codes	Frame and category	Codes
POWERLESSNESS Vulnerable 34%	Dependent Frail Physically weak Sedentary Slow Weak Victim Vulnerable	HUMAN IMPACT Mentor/ Role Model 17%	Experienced Defies perils of aging Someone to laugh with Mentor Revered Role Model Survivor War Veteran/Hero Wise
POWERLESSNESS Declining 21%	Declining Forgetful Grumpy Senile Stupid Ugly Unhealthy Wrinkly Boring	HUMAN IMPACT Perfect Grandparent 5%	Caring for children Kind Practiced Perfect Grandparent Sense of humor Smiling Warm
POWERLESSNESS Burden 19%	Burden Demographic time-bomb (global graying, silver tsunami, boomer) Societal drain Non-contributor Past-it Obsolete Family problem	HUMAN IMPACT Golden Oldie 4%	Active Affluent Golden Oldie Happy Leisure-oriented Youthful

### Central Narratives of the Elderly Framed Negatively

A clear majority of phrases (74%) used the dominant framing of powerlessness by positioning “the elderly” as a weak or vulnerable group in relation to prevailing societal groups. “Elderly” was constructed individually, or as a group, as vulnerable, frail, declining, and a burden in a partly or entirely negative manner. Findings are reported here with corresponding numbers to indicate the source of each quote (readers are reminded to refer to the data extract in Appendix 1). Many phrases directly linked the elderly with being vulnerable, e.g., “Firms seeking to make ends meet will cut back on staff and the truly vulnerable, the elderly, will suffer.”^1^ and “Our most vulnerable—the elderly, the very young, migrants, ethnic minorities, the socially isolated and renters—would be hardest hit,”^2^ and “The wellbeing of a nation is shown by how it values its weakest most vulnerable members—children, the sick, the elderly, the poor and the unborn.”^3^

These latter two phrases illustrate another common approach, which was pairing “the elderly” with other marginalized groups, including babies, children, pregnant women, the disabled, the frail: “*Person* expressed concerns about vulnerable users riding the e-scooters or being hit by others, including the elderly, children and parents with young children,”^4^ and “They say cancelled [bus] trips and lack of shelters have left the community’s most vulnerable users—children, the elderly and people with disabilities—at risk.”^5^

The vulnerable narrative was often substantiated through the inclusion of statements from those considered to be experts (i.e., medical practitioners, aged residential care professionals, police), who were quoted discussing apparent risks to all “elderly.” The following examples function to further strengthen and legitimize media positioning of the “elderly” as vulnerable or a burden: “The frail, elderly and young were the most affected by the heat, *expert* said”^6^ and “*Professional* says that the Christmas and holiday period can be a time of intense loneliness for our elderly”^7^ and “"We urge people to have conversations with vulnerable or elderly family members, to help ensure they are aware of the tactics often used by scammers and don't become victims," says Detective Sergeant.”^8^

Another narrative clearly emerging was “elderly” as a burden (19%), whether for individuals, communities, or society at large. Phrases such as this, “There are elderly people with walking frames, people with mobile scooters, mothers with pushchairs and prams—they all create some sort of a hazard”^9^ not only depict “elderly” as vulnerable or frail through connecting them with “walking frames,” but also perpetuate the burden stereotype through use of the word “hazard.” “Elderly” was often represented as a burden on society generally, or the health care or financial system. Examples include, “My only disappointment was no mention of inheritance tax if you [move away] to live in [Australia] and expect the rest of us to pay for your elderly relatives,”^10^ and “Does he think the frail elderly are expendable? Perhaps we could expand his thinking to a way of freeing our society from the burden of care for them by planting COVID in every retirement facility.”^11^ and “They will be paying higher taxes due to the debt, rising unemployment, and the cost of supporting the elderly on National superannuation.”^12^

Many phrases illustrated how older adults have internalized the burden narrative: “…for some elderly the thought of asking family or friends to do their housework was out of the question. They don’t want to be a burden. Sad but true.”^13^ and “Some elderly people are so concerned about leaving a legacy of debt that they sacrifice their standard of living to cover the cost of their death.”^14^ Media use of such phrasing reinforced how individuals self-direct ageism through a process of cultivation (Gerbner, 1998) and shape their behaviors accordingly.

Terms such as “boomer remover”^15^ and “silver tsunami”^16^ were also used in connection with “the elderly,” presenting a contemporary manifestation of a long-standing tradition of perpetuating ageism through language choice, rather than an occurrence unique to the present age. In conjunction with this, the contrast between youth (e.g., millennials) and “the elderly” (e.g., boomers) also encouraged the view that “the elderly” is a group presenting an economic, health and societal drain, a burden more so now because of population aging. Such statements risk promoting an intergenerational warfare narrative (Morgan et al., 2021) where the elderly are a burden on younger, economically engaged individuals, as these quotes express: “New Zealand’s aging population is putting pressure on the health services for the elderly”^17^ and “This [COVID] isn't about Millennials saving Boomers, it's about the country banding together to save itself, experts say”^18^ and “The fact that aged care funding has not yet become a crisis speaks volumes for the people who care for our frail elderly, but their goodwill cannot be inexhaustible.”^19^

Although many phrases may have appeared to be respectful to “the elderly,” the stance was undermined by adding a ‘but,’ as illustrated in this phrase: “*Person* is a character that tugs on the heartstrings. He’s everybody’s elderly granddad, frail but stubborn, confused and endearing.”^20^ Such wording implies that this character is appealing, but powerless. These and other phrases like “Tuvaluans, they have different types of months, not like January to December,” said the elderly but agile man, who still climbs coconut trees every day^21^ and “It’s not very often that elderly New Zealanders attract our attention, but COVID has seen the plight of some of our elderly people in the headlines for weeks”^22^ highlight how “elderly” is framed using both the powerlessness and the human impact frames which invites the audience to buy into the negative and positive stereotype dichotomy. In data where “elderly” was framed powerlessly through negative stereotypes, there were three overarching categories identified: vulnerability, declining, and burden.

### Vulnerability

Vulnerability (34% of all data) and weakness were frequently expressed by pairing the term “elderly” with other population groups including infants, children, disabled, poor, pregnant women, or often with the word vulnerability itself: “*Person* says of our elderly and vulnerable families who received support, many came to tears as they were handed their parcels of food, so deep were their feelings of appreciation that strangers actually cared for them.”^23^ This is not a minor finding. “Elderly” was largely characterized as vulnerable or victims in ways that encouraged sympathy (or pity) within the reader: “Self-isolation means many of our most vulnerable are struggling to access essential services. We have heard of an elderly lady who had not left her home for days and had run out of food.”^24^

In subtle and not-so-subtle ways, such phrasing had the effect of disempowering and differentiating “elderly” from the main population, implying that everyone in society form the in-group, but “the elderly” form an out-group (frequently paired with other marginalized out-groups), e.g., “*Politician* agrees, but said he will keep in touch with what’s happening, and vote for any amendments that give more protection to the elderly and vulnerable.”^25^ The practice of othering through negative stereotype use in online news articles delineated an in-group (non-elderly, who hold the power) and an out-group (elderly, who are powerless). Of specific relevance here was the partiality to negatively stereotype “elderly” with reference to their alleged vulnerability, further constructing and reflecting this group as the out-group who are least able to resist any changes to the status quo.

### Declining

Stereotypes of decline (21%) were evident in messages that cast elderly as declining bodily, being grumpy, forgetful, rude, or losing their status or position in society: “So we are limited, at best, to a description of an elderly woman, with no teeth, of slight build”^26^ and “It was not possible for a small group who were elderly, very conservative and had been powerful in the past to shift a whole society."^27^ Descriptions of grumpy or stupid largely cast “elderly” as declining through word choices that constructed either single individuals as frail or “losing it” e.g., “An elderly man who harassed and threatened a neighbor over her dogs, which he claimed kept him awake, has been ordered by a court to stay away from her for five years,”^28^ or a mob who should know their place, e.g., “Lately though, it is the elderly who seem to be the nastiest. They should know better.”^29^ Association with physical, mental, and social decline frames “the elderly” as powerless, lacking full capacity (“Ultimately, there is the question of the cost and inconvenience that families and the state are prepared to bear, to provide such mechanisms, in light of the other demands in caring for the elderly”^30^) and those responsible for looking after or caring for “the elderly” as righteous and charitable in their motives.

### Burden

Almost one-fifth (19%) of data framed “elderly” as a burden, warning readers of the supposed problem of “the elderly” as expressed by this headline: “Grey Tsunami Warning.”^31^ Negative stereotypes communicated that as people age, they become obsolete, no longer of use and simply a burden at societal, community, and individual family levels. “Grave economists, actuaries, statisticians, and politicians by the score—mostly young, you'll note, will heat much air, in rousing fears and giving vent on Twitter, in Parliament and the press—on just how costly it's become to house, and feed, or nurse the elderly in their declining years.”^32^

Being positioned as a burden was in connection with financial implications for pensions and taxes, using up healthcare resources that could otherwise be available for the rest of society, for instance, but also inconveniently relying on family members for individual care: “This is a reluctant sale but *person* says she needs to be closer to her elderly mother who lives across the Harbor Bridge”^33^ and “I hope your parents and/or grandparents don’t need support. I have an elderly and very frail lady as a neighbour.”^34^

### Central Narratives of the Elderly Framed Positively

Fewer data used the secondary framing of human impact and framed “the elderly” more optimistically by using positive stereotypes (26%). Positive stereotypes were identified across three over-riding stereotypes: mentor/role model (17%), perfect grandparent (5%), and golden oldie (4%).

### Mentor/Role Model

Among the positive stereotype data, the notion was present in the Mentor/Role Model category (17%) that “the elderly” are valued for their experience, can offer wisdom, and act as role models within society. Take, for instance, “Whatever the case, no-one was seriously injured as elderly bystanders threw themselves in to hold the scuffling parties apart"^35^ and “Thumbs up to the elderly lady with the walking stick for picking up the rubbish along the lake shore at the lake front on Sunday.”^36^ Although this phrase could be seen to cast “elderly” as declining through use of the description “walking stick,” it also paints a picture of “elderly” doing the “right” thing and being a role model in society. In this case, while the initial description of the elderly lady was constructed as declining, the positive act she was undertaking resulted in people taking notice, which further highlights the supposed duality of “elderly” as both wise and weak.

Being called “elderly” was also constructed as having potentially positive outcomes for individuals or groups, e.g., “[The ball] is in honor of the elderly. It is really important to acknowledge our elders and what they have done for us in the community.”^37^ In this category, the term “elderly” was used in conjunction with war veterans, who were revered or elevated to a celebrity-like status. Similarly, data analysis illustrated centenarians were likened to heroes.

### Perfect Grandparent

A second narrative among the positive stereotypes was the Perfect Grandparent (5%), also reported by Ylänne (2021) about media representations of aging. Stories using “elderly” in relation to grandparenting implied that all elderly were kind, loving, family-oriented, “Grandparents can safely hug under-10 s”^38^ and willing to volunteer their help and put the younger generation ahead of themselves and their needs: “Your father sounds like a real character and your boys are lucky to have an elderly person in their lives who loves them and is prepared to teach them things.”^39^ However, the same article carries on to also state, “There's a trade-off though, as he offers all that good stuff elderly people bring to the table, but your wife doesn't think his supervision is up to standard.”^40^ As the last phrase illustrates, somewhat overlapping with the other narratives, the supposed duality of “elderly” as both a wise mentor (all that good stuff) and an old fool (supervision of children is not up to standard). Phrases referring to grandparents were described in mainly positive personality-related terms and used the human impact frame (Neuman et al., 1992).

### Golden Oldie

Lastly, again framed through human impact, the Golden Oldie (4%) category referred to stereotypes of “the elderly” being affluent in retirement, being active, enjoying the post-working years through their orientation to leisure. “My generation really did live through a remarkably successful economic period and many of us are now comfortable and able to enjoy our retirement…”^41^ Positive stereotypes identified “the elderly” as experienced, people to laugh with, caring for children, being a survivor and being financially comfortable, active, warm, leisure oriented: “*Organizer Name* and several volunteers brought the equipment and provided the transport, so elderly participants in the "blokes' day out" just had to sit back and fish from Nelson's Sunderland Marine Pier.”^42^

### Central Narratives of COVID

**Fig. 1:**
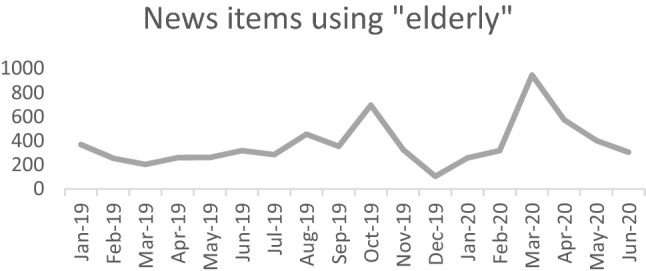
News items containing “elderly” recorded by month Jan 2019–Jun 2020

Whether articles were an opinion piece, a feature article, report, letter to the editor, or a newswire, stories clearly clustered around five recurrent topics (and were largely covered superficially): housing, transport, health, finances, and COVID. It is beyond the scope of this article to present detailed data for all five story topics; however, the most contemporary findings related to the COVID pandemic are summarily reported here. Figure 1 illustrates the dramatic rise in number of references to the “elderly” during the month of March 2020, which coincided with the first pandemic lockdown in New Zealand.

During the first twelve months of January to December 2019, “elderly” appeared an average of 324 times per month, or approximately 10.8 times per day. A small spike in October 2019 possibly related to national and political conversations occurring around dementia care and aging populations. Usage fluctuated across four over-riding story topic areas (housing, transport, health, finances) in a relatively even manner. Even in December 2019, when the COVID pandemic broke out in China gaining world attention, and continuing into January and February 2020, numbers were lower (an average of 227 times per month). This could be attributed to the summer vacation and 6-week Christmas school holiday period.

Dramatically, in March 2020, numbers rose to 946, with close to 87% of usage relating to the COVID pandemic. Results showed that particularly during March 2020, homogenization was ever present, creating the “elderly” as *the most vulnerable* group during the pandemic, overwhelmingly framed as being at risk and passive, with no regard for diversity of social situations, intersecting identities, including ethnicities. For instance, this phrase in the Taranaki Daily News 16 March 2020 captures the sentiment well: “Ardern asked Kiwis yesterday to practice as much "social distancing" as possible—putting off visits to elderly relatives, forgoing handshakes or hugs, and staying home from work if unwell” (Social distancing was then an unfamiliar term). Flett (2020) found that media coverage plays a direct role in shaping official responses to crises and helps to convey who matters most (and thus, who are valid recipients of governmental or community support). In this way, media contributed to social control of an already structurally disadvantaged group.

In all, the central narratives, and discourses of “elderly” in relation to COVID, served to position older adults as vulnerable, at risk, and susceptible to dying from the virus more so than any other group in the population. This is not to minimize the seriousness of the virus for adults in later life. It is to point out how the pandemic crisis affected older adults psychologically, emotionally, and socially as media reflected and perpetuated the “burden” and the “intergenerational warfare” narratives (e.g., boomer remover^15^) by strongly casting “elderly” as vulnerable and weak.

## Discussion

This research used a critical gerontological framing analysis to interpret a sample of 6,690 phrases containing “elderly” found in New Zealand online news media during 2019 to 2020. In many ways, the results may not be surprising overall, but they are nonetheless disturbing. Portrayals of older adults as “elderly” reflected a narrow and negative image of vulnerability, decline, dependence, and being a burden in around three-quarters of the data. “Elderly” was frequently othered; often paired with babies, children, pregnant women, and “the disabled,” or “the vulnerable.” Only one-quarter of data conveyed positive depictions, but most of these tended to reinforce stereotypical, narrow views through sentimentality (see Binstock’s, 1985 compassionate ageism). Using positive stereotypes to link “elderly” with being role models, golden oldies, or perfect grandparents tends to ignore economic, cultural, gender, and racial diversity among older adults by homogenizing them all as middle-class, in nuclear, heterosexual, familial contexts.

### Implications for Adult Development Within Cohorts

How “the elderly” is framed in the media has implications for how adults develop. Messages are often internalized and influence how individuals of all ages view themselves, others, and how they behave (Fraser et al., 2016; Gilleard & Higgs, 2007; Wiles et al., 2012) in a form of self-directed ageism. “Self-directed ageism refers to ageism turned against oneself. People internalize biases based on age from the surrounding culture after being repeatedly exposed to them, and they then apply the biases to themselves” (WHO, 2021, p. 166).

Continued media coverage using this term may contribute to older New Zealanders feeling socially excluded (Wiles et al., 2012) and induce others, especially younger generations, to begrudge older citizens’ worthiness and entitlement to receive national support and goodwill (Flett, 2020). At a wider level, consequences of ongoing unchallenged ageist stereotypical perceptions towards “the elderly” do little to encourage empathy and solidarity between older and younger adult generations. It may even contribute to intergenerational conflict. As Vasil and Wass (2006) predicted, ageism is becoming a significant social issue the more the ratios of generational groups shift due to aging populations (Kreibernegg, 2018). Depictions of “elderly” displaying agency in interpersonal relations were notably absent from the stories analyzed in this study, having implications for intergenerational relations.

Interviews with older adults themselves were largely absent. Critical issues pertaining to the importance of life-long education, work and family changes, physical and mental health influencing development were barely visible. For instance, life course transitions of retirement, divorcing and re-partnering, negotiating everyday life with memory loss (Beard & Fox, 2008), music, sports, business, or older adults’ views on the present, or stories from the past were largely absent. Kovács et al. (2021) suggest that under-representation or invisibility conveys that older people are less important, less worthy of media attention than other age groups, or non-contributing, non-participators in society.

Viewed from a critical gerontological perspective, these research findings point to a need to shift the media view that old age is an end point—an older adult is still journeying through their life course. Just as with younger generations, development of personality, emotions, cognition, and biomarkers continue to be areas of interest for older adult development.

### Deconstructing “Elderly”

Katz (1996) proposes that “The creation of the elderly as a population must be seen against the historical background of demographic discourse itself” (p. 127), a discourse of ‘crisis of capacity’ which originated in the nineteenth century. A discourse of crisis and burden produces the “elderly population” as a distinct group that threatens social and economic stability (Kreibernegg, 2018). Creation of an “elderly population” is further premised on inherent assumptions that youth and agility comprise a worthy life, while vulnerability, weakness, disability, and frailty comprise a worthless life (Kreibernegg, 2018; Pack et al., 2019). “Elderly” represents the other, embodying decline, decay, and death which must be excluded from everyone else and put to the side of “normal” life to avoid fear, dread, or alarm. Like all othering, “the elderly” is based on a discourse of exclusion through its binary construction.

The term “elderly” itself is a specific example of how older adulthood is constructed on a binary of inclusion and exclusion in the New Zealand context. Findings may well be applicable in other countries. Coupland (2009) states that “people are simultaneously the products and the producers of discourse” (p. 856). Societal othering of older adults is not a recent phenomenon (Kreibernegg, 2018). Language may change over time to achieve such goals, e.g., “boomer remover”^15^ or “silver tsunami”^16^ are examples of language that both reflect and construct the context of aging experiences throughout adulthood across diverse cohorts. Such phrases risk building an intergenerational warfare narrative (Morgan et al., 2021) and perpetuating discourses of older cohorts being a burden on younger, economically engaged adults. Binary constructions through dichotomous negative and positive stereotypes have potential to influence, shape, and *cultivate* (Gerbner, 1998) societal and individual perceptions and behaviors.

### Future Research

Future research must respond to a growing intergenerational conflict focused on the “burdensome” nature of societal aging to explore outcomes and impacts of using more accurate representations in online news media of the wide range of aging trajectories. In the present research, data collection and analysis were restricted to a New Zealand context—this is a limitation. More research is needed to confirm the outcomes and impacts of using positive aging stereotypes in online news media in wider contexts. It is possible that the findings from this research could apply to other countries and cultures.

Superficially, positive, or successful aging stereotypes and attitudes could negate traditionally damaging stereotypes and attitudes and begin developing agentic spaces for older adults. Nevertheless, critical gerontological examination of these discourses draws attention to the disassociation of aging and the underlying pursuit of never-ending youth as part of these stereotypes (Pack et al., 2019). Therefore, Pack et al. (2019) outline an existing counter argument that positive aging discourses are *also* a repudiation of being old, a rejection of bodily decline, and have the outcome of reproducing and perpetuating ageism. In all, the critical framing analysis of this study indicated that both negative and positive stereotypes produce othering effects by socially creating a dominant in-group of non-elderly and a minority out-group of “the elderly.”

### How Can Media Contribute to Changing the Narrative?

This article has focused on ageism towards older adults—that is institutional, interpersonal, and self-directed—serving to highlight the importance of interindividual differences and contextual issues that influence older adult development. Only by gaining a deeper understanding of how terms like “elderly” betrays ageism can the seriousness and problematic impact for older adults’ development be understood. By learning to identify systemic ageism, online news media organizations could join and contribute to global efforts (UN, 2021) that are encouraging governments to address and respond to ageism. Media organizations are well positioned to raise awareness of and build understanding in communities about what ageism looks like, and why it needs to be a priority to challenge negative narratives.

Social responsibilities of news media to report news that inform and serve the needs of all citizens involve presenting stories in ways to address their complexity and diverse perspectives. Online news media organizations operate in a competitive environment that demands instantaneous news delivery. In these pressured contexts, responsibility to ethically represent particular social groups is, at times, in direct tension with bottom-line economic concerns (Patterson et al., 2019). While traditional print media tended to be premised on objectivity of journalists, online news media has brought about a wider concern (Paulussen & Ugille, 2008), and to some degree, acceptance (Canella, 2021; Ward, 2010) that a reporter’s personal subjectivity, experience, culture, and beliefs influence how they perceive issues.

These findings present online news media reporters and publishers with an opportunity to intentionally use language that reflects and produces more helpful societal attitudes among New Zealand citizens. Canella (2021) proposes that as a guiding principle for journalism practice, objectivity has declined in favor of more “opinionated and emotive journalism” (p. 3), while Ward (2010) presents the concept of “pragmatic objectivity” which embraces a journalist’s subjectivity and acknowledges the interpretive practices used to produce news like interviews, judgments, and checking methods. Evidence from this study suggests that New Zealand online news media have more to do to meet their social responsibilities of using respectful language when reporting about older adults. Professional development and more media training about less ageist language are useful starting points. Particularly, going forward in a COVID context, messages concerning older adults are important to achieve age equality and avoid discriminatory behaviours (UN, 2020). Actions of othering perpetuate discourses of aging as being problematic and that old age must be rejected, decelerated, or hidden.

## Conclusion

The aim of this study was to understand specifically how the term “elderly” is used in online news media articles in New Zealand presently. The study confirms and adds to findings from previous studies on older adults in the media and in the news. Results of this research found that groups of older adults perceived to be “elderly” in New Zealand were negatively stereotyped in three-quarters of the data through associations with vulnerability, decline, and being an individual or societal burden to others.

Researchers (Gendron et al., 2016) argue that words or phrases referencing older adults must be carefully selected or risk homogenizing all older adults in terms of their “health, capabilities, dispositions, desires, socio-economic status and social needs” (p. 100). The phrase “the elderly” is a linguistic generalization. Associated stereotypes of “the elderly” identified in this study give rise to stigmatism and sustained ageism towards older people, impacting adult developmental trajectories through exclusion and marginalization. Without appropriate steps being taken, endemic ageism will persist unless we all respond to it.

New Zealand society needs to gain greater understanding of all aspects of ageism and how to recognize, reduce, and reject it. Interventions that promote optimal development throughout adulthood, and especially older adulthood are often sought in health fields, however, media intervention in this journey towards age equality is welcome. For media efforts to be successful in changing how New Zealanders think, feel, and act towards age and aging, three recommendations have become evident from this research that media can take to help New Zealanders make the change. First, participate in active approaches to avoid and act towards ageism. Second, invest in staff training and knowledge-building to improve on identifying and addressing ageism. Third, build a commitment to changing the narrative around aging and old age. Looking globally to encouraging efforts such as the *Reframing Aging Initiative* (2022) in the US and the *Centre for Ageing Better* in the UK (2022) can also provide useful information and resources for specific journalism techniques.

Within and likely beyond New Zealand, online news media articles need to replace the term “elderly” with “older adults,” “or “older people,” or as advocated by the UN (2020), “older person/s.” Findings from this study point to online news media taking responsibility to act upon this immediately and indicate they are on the journey to age equality. Considering the intergenerational relationships among diverse adult cohorts going forward, alongside effects of the continuing COVID pandemic and an increasing reliance on digital technology, online news media has a key role to play now more than ever.

The term “elderly” is ageist and should disappear from online news media reporting. Replacing this word with more respectful language opens the way forward for promoting non-discriminatory, open-minded societal views of equality in aging. Bringing about a focus on the uniqueness of individuals and having the courage to challenge disrespectful practices by removing the term “elderly” from online news articles is one way that media could help all global citizens to promote healthy adult development as we age.

## Appendix: Example of Data Reported in Results

**Table:**

	Source	Date	Article Type	Title
1	Stuff-Sunday Star Times	Apr 2019	Opinion	A lesson from the aged care workers
2	Herald on Sunday	Sep 2019	General News	Health issues are heating up
3	The Daily Post Saturday	Oct 2019	Regional News	Parents, teachers, health staff make abortion bill input
4	New Zealand Herald	Feb 2019	Sports News	Lime warned its e-scooters could be hauled off streets this week
5	Bay of Plenty Times	Feb 2019	Regional News	Getting riders back on board
6	Bay of Plenty Times	Jan 2019	Weather News	Tauranga temperatures soar with more people showing up to hospital…
7	Scoop	Dec 2019	Culture, Newsworthy	Aged care expert’s top tips for a Merry Christmas
8	Scoop	May 2019	Regional, Crime	Don’t go breaking your bank by falling for scams
9	Nelson Mail	Apr 2019	Regional	Tasman to host Paxster trial
10	Nelson Mail	Feb 2019	Opinion	Tax system
11	The Dominion Post	Mar 2020	Letter to Editor	Only the frail elderly
12	The Dominion Post	May 2020	Letter to Editor	Level 1 and taxes
13	Hawkes Bay Today	Jan 2020	Editorial Opinion	Middle NZ: Good our DHB has listened
14	The Dominion Post	Jul 2019	Feature	Our $42 m bill for funerals
15	News Hub	Mar 2020	General News	Coronavirus: COVID’s controversial new social media nickname
16	The Northland Age	Feb 2019	Regional News	Planning urged for growing ‘silver tsunami’ approaching
17	Sunday Star Times	Feb 2019	Business News	Boomers want to die at home
18	Stuff.co.nz	Apr 2020	National News	This isn't about Millennials saving Boomers, it's about the country banding together to save itself, experts say
19	The Northland Age	Jun 2020	Editorial	The big health issues
20	CHB Mail	Apr 2019	Culture, Regional News	Homeland tugs on heart strings
21	Radio NZ Newswire	Oct 2019	Climate Report	Ocean 'breaking point': Pacific angst at latest climate report
22	The Northland Age	Jun 2020	Opinion	A taste of old age
23	Waikato News	Jun 2020	Community News	Helping our homeless, isolated, old, unemployed
24	Manawatu Standard	Mar 2020	Regional News	Help to aid welfare launched
25	Whanganui Chronicle	Jul 2019	Political News	Our MPs opposed to Euthanasia
26	Hawkes Bay Today Sat	May 2019	Regional News	The Hawke’s Bay’s Earthquake’s Jane Doe
27	Stuff – The Press	Feb 2019	National News	National Portrait: *Name*, Treaty Historian
28	The Dominion Post	Mar 2019	District Court News	Barking leads to restraining order for neighbor
29	Daily Post	Apr 2019	Opinion	Health and safety matters should not be trivialized
30	Otago Daily Times	Aug 2019	Regional Opinion	Ways to decide needed for people lacking full capacity
31	The Daily Post	Jul 2019	Regional News	Grey tsunami warning
32	The Dominion Post	Jul 2019	Letter to Editor	Swings and roundabouts
33	Wed Herald Homes	Apr 2019	Feature	Teeing up view of golf course
34	Hawkes Bay Today	Jan 2020	Letter to Editor	Forget DHB 'fairyfloss,' we'll all be old one day
35	Dominion Post	Jun 2020	Report	Prayers and a punch up
36	Taupo Weekender	Jul 2019	Community News	Thumbs up and down
37	Horowhenua Chronicle	Feb 2020	Regional News	Live band ready for Foxton Ball
38	The Dominion Post	Apr 2020	General News	Grandparents can ‘safely hug under-10 s’
39 and 40	Stuff.co.nz	Sep 2019	Lifestyle/Parenting	My wife isn’t happy about my dad’s grandparenting style
41	Bay of Plenty Times	Feb 2020	Financial News	Brace for a big shift
42	Nelson Mail	Jan 2019	Regional News	Fishing trip helps elderly hook up with friends
